# Tools for Identifying and Prioritizing Evidence-Based Obesity Prevention Strategies, Colorado

**DOI:** 10.5888/pcd10.120275

**Published:** 2013-06-27

**Authors:** Gabriel E. Kaplan, Ashley L. Juhl, Indira B. Gujral, Andrea L. Hoaglin-Wagner, Barbara A. Gabella, Kristin M. McDermott

**Affiliations:** Author Affiliations: Ashley L. Juhl, Indira B. Gujral, Andrea L. Hoaglin-Wagner, Barbara A. Gabella, Kristin M. McDermott, Colorado Department of Public Health and Environment, Denver, Colorado.

## Abstract

Colorado’s adult obesity rate has more than doubled since 1995, prompting its Department of Public Health and Environment to list obesity as its top prevention priority. To initiate comprehensive and effective action, the department used a well-known evidence-based public health framework developed by Brownson and others. This article describes the tools and process developed to conduct 2 of the 7 stages in this framework that challenge public health organizations: reviewing the literature and prioritizing effective strategies from that literature. Forty-five department staff participated in an intensive literature review training to identify physical activity and nutrition strategies that effectively address obesity and worked with external stakeholders to prioritize strategies for the state. Divided into 8 multidisciplinary teams organized by the setting where public health could exert leverage, they scanned the scientific literature to identify potential strategies to implement. These teams were trained to use standardized tools to critique findings, systematically abstract key information, and classify the evidence level for each of 58 identified strategies. Next, departmental subject matter experts and representatives from local public health and nonprofit health agencies selected and applied prioritization criteria to rank the 58 strategies. A team charter, group facilitation tools, and 2 web-based surveys were used in the prioritization stage. This process offered the staff a shared experience to gain hands-on practice completing literature reviews and selecting evidence-based strategies, thereby enhancing Colorado’s obesity prevention efforts and improving public health capacity. Practitioners can use these tools and methodology to replicate this process for other health priorities.

## Introduction

A scientific approach to the formulation of evidence-based public health strategies was advanced as a way to improve the performance of public health departments and make the best use of limited resources (1). Evidence-based public health (EBPH) is defined by Kohatsu as “the process of integrating science-based interventions with community preferences to improve the health of the population” (2). However, constraints and challenges, ranging from limited time to insufficient skill with evidence-based methods, make the real-world implementation of this approach difficult (3). At the state and local level, trying to follow scientific approaches is often impeded by day-to-day realities of conducting public health.

The EBPH framework by Brownson et al (1) is a 7-stage process (Figure) that combines research and surveillance evidence with the needs, values, and preferences of the population (1,4). It offers a systematic and rigorous way to approach the complex tasks of identifying problems, marking leverage points, and implementing the most effective and promising solutions. However, each stage can require thoughtful and skilled application of social scientific methods. The multidisciplinary backgrounds and responsibilities of the public health workforce can mean that staff members lack the skills, confidence, or time to conduct each stage.

**Figure:**
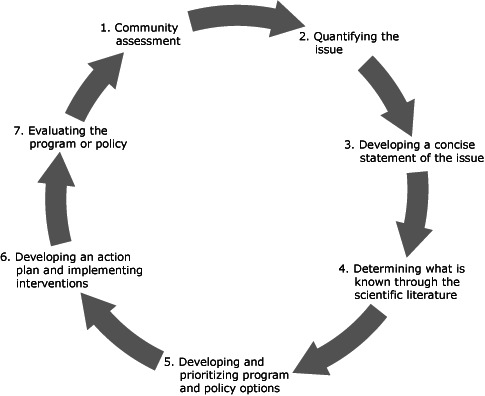
Evidence-based public health framework (1). The 7 stages of the evidence-based public health framework.

The stages in Brownson’s EBPH framework connect existing skills and practices of public health entities, such as conducting surveillance, analyzing epidemiologic evidence, drafting work plans with SMART (specific, measurable, attainable, relevant and time-sensitive) objectives, and instituting evaluation designs. Of the 7 stages, 2 can pose operational challenges: determining what is known through the scientific literature (stage 4) and developing and prioritizing program and policy options (stage 5).

The Colorado Department of Public Health and Environment (CDPHE) applied the 7-stage evidence-based public health framework to the topic of obesity prevention and control. Although Colorado ranks lowest among the states in adult obesity prevalence, it has not escaped the burgeoning epidemic. Adult obesity prevalence nearly doubled from 1995 to 2010: (10.7% in 1995 to 19.8% during 2008–2010), and Colorado’s child obesity prevalence is in the middle of state rankings (5). CDPHE and other public health entities are challenged by gaps and changes in the evidence about what works (6). Therefore, by reviewing the latest evidence across a greater array of potential interventions than just those available in the *Guide for Community Preventive Services*, CDPHE could select strategies that align with the evidence.

Armed with epidemiologic data and a clearer understanding of the obesity issue as it relates to Colorado (stages 1–3 of the framework), CDPHE embarked on a 6-month planning process to review the literature and prioritize strategies (stages 4 and 5) beginning in December 2011. The objectives were to identify physical activity and nutrition strategies to reduce obesity, rate the level of evidence supporting their effectiveness, set priorities for the rated options, and select priority strategies for implementation that promise the most efficacious approach for Colorado. For this process, strategies were broadly defined as “public health interventions, methods, approaches, or programs to increase physical activity and healthy eating.”

The purpose of this article is to describe the tools and planning process that 1 state developed to conduct these 2 challenging stages in the Brownson et al evidence-based public health approach (1): to review the literature related to obesity strategies (stage 4) and to prioritize effective public health strategies for Colorado (stage 5).

## Determining What Is Known Through the Scientific Literature (Stage 4)

The goal of stage 4 is to determine what is already known about the effectiveness of strategies through the scientific literature, which includes systematic reviews, published research, and high-quality evaluation reports. A common but labor-intensive method is a formal literature review or systematic approach to identify, access, and assess the relevancy and quality of the research.

To conduct a large, comprehensive literature review in a short time, the planning process organizers tasked approximately 45 staff members from units that focus on chronic disease, injury prevention, physical activity, nutrition, and child and women’s health to conduct the review. The organizers grouped them into 8 multidisciplinary teams (Table 1). Each team focused its review on a different setting where CDPHE had influence: the built environment, child care, community, food systems, health systems, media, schools, and worksites. Each team designated a lead and had an epidemiologist or evaluator to assist throughout the literature review.

**Table 1:** Working Groups for Stage 4 (Literature Review) and Stage 5 (Prioritization) of the Evidence-Based Public Health Framework^a^, Colorado, 2011

Working Group	Stage	Membership	Responsibilities
8 Literature review teams (1 for each of 8 settings where an intervention could be implemented).	4	45 members from CDPHE working in the fields of chronic disease, injury, child health, and women’s health	Search, review, and rate the literature about increasing physical activity and nutrition in a particular setting and use/complete tools.
Planning committee	5	5 members: 3 internal CDPHE staff and 2 external facilitators.	Develop prioritization tools; plan and facilitate meetings of the steering committee.
Steering committee	5	20 members: 12 CDPHE leaders from literature review teams; 5 local public health agency representatives; 3 external stakeholders.	Select prioritization criteria; prioritize 58 strategies for the Executive Committee.
Executive committee	5	6 members: managers from CDPHE’s Prevention Services Division, chronic disease epidemiologist, obesity expert.	Select final strategies for adoption and implementation by Implementation Teams.
Future implementation teams	6	CDPHE staff and external stakeholders.	Plan and implement prioritized obesity strategies.

Abbreviations: CDPHE, Colorado Department of Public Health and Environment.

a Brownson et al. (1).

Staff participated in a 2-day hands-on training and implementation workshop to learn how to identify effective physical activity and nutrition strategies for obesity prevention from the scientific literature. Created and led by 2 epidemiologists, the training instructed staff on evidence-based public health, searching the literature, accessing full-text articles, critiquing articles, and rating the evidence that a particular strategy will be effective for obesity prevention. The large number of staff involved in the process allowed the teams to look at a large number of articles and reviews, and the training gave the staff a base level of knowledge. Instruction on how to conduct literature reviews was also intended to build capacity among staff members who could then integrate literature reviews into their own work.

The training for the literature review and rating used 4 tools to organize and move the 8 teams through the assessment process (Table 2). Two tools were introduced during the first training and the other 2 during the second training. The Literature Review Critique tool guided participants through abstracting pertinent information from systematic and peer-reviewed articles. The Literature Library tool helped participants organize the articles reviewed. As instructed at the end of the first training, the teams used the 4 weeks between the 2 trainings to complete their literature searches, critique the articles, and organize them in the Literature Library.

**Table 2:** Tools Developed and Used for Literature Review

Tool	Description
Literature Review Critique	A 13-item form with questions to consider when reading an article. Includes questions regarding the article source, introduction, methodology, results, and discussion (http://btfy.me/yg3mt9).
Literature Library	A table on which to enter information extracted from each article used in the literature review. This table has space to describe article, methodological characteristics, results and conclusions, notes from critique, and strategy type (http://btfy.me/h3b4md).
Summary of Evidence	This tool has 3 sections: the literature inventory, the classification of evidence, and the state-level implementation score. One summary is completed for each strategy (http://btfy.me/hcryb6).
Typology for Classifying Interventions and Strategies by Level of Scientific Evidence	This tool (described in Table 3) describes the 5 levels used to rate the evidence. It gives examples of how the rating is established, what to consider for each rating, and data source examples (http://btfy.me/xy6rtw).

The first day of training focused on searching the literature and critiquing the articles. The instructors taught participants about 3 types of literature (systematic reviews, peer-reviewed journal articles, and grey literature) and demonstrated how to use selected databases to search for suitable articles, including The Cochrane Library (http://www.thecochranelibrary.com), The Campbell Library (http://www.campbellcollaboration.org), The Guide to Community Preventive Services (http://www.thecommunityguide.org), PubMed (http://www.ncbi.nlm.nih.gov/pubmed/), Science.gov (http://www.science.gov/), and The New York Academy of Medicine Grey Literature Report (http://www.nyam.org/library/). The instructors showed staff how to use the PICO (population, intervention, comparison intervention, and outcome) methodology to create search questions (7). Although some articles were immediately available through PubMed Central, the teams accessed many articles through the CDPHE Digital Library, a pilot project of the National Network of Libraries of Medicine (http://nnlm.gov/) that allows state-level staff direct access to full-text resources.

The second day of training focused on rating the literature and summarizing the evidence for each identified strategy. The instructors introduced a tool called the Summary of Evidence, and the teams populated the form with information about each obesity strategy. The teams then used the Typology for Classifying Interventions and Strategies by Level of Scientific Evidence (Table 3) to assign an evidence rating to each strategy. CDPHE adapted this typology from *Healthy People 2020*’s version (8), which in turn had adapted it from a typology by Brownson et al (1). At the end of the second day, each team reported its evidence ratings for each strategy (58 in total) to the other teams.

**Table 3:** Typology for Classifying Interventions and Strategies by Level of Scientific Evidence

Level of Evidence	How Established	Considerations for Level of Scientific Evidence	Data Source Examples
Proven	Peer review via systematic or narrative review	Based on study design and execution External validity Potential side effects or harms Costs and cost-effectiveness	Community Guide Cochrane reviews Narrative reviews based on published literature
Likely Effective	Peer review	Based on study design and execution External validity Potential side benefits or harms Costs and cost-effectiveness	Articles in scientific literature Research-tested intervention programs Technical reports with peer review
Promising	Written program evaluation without formal peer review	Summative evidence of effectiveness Formative evaluation data Theory-consistent, plausible, potentially high-reaching, low-cost, replicable	State or federal government reports (without peer review) Conference presentations
Emerging	Ongoing work, practice-based summaries, or evaluation works in progress	Formative evaluation data Theory-consistent, plausible, potentially high-reaching, low-cost, replicable Face validity	Evaluablility assessments^a^ Pilot studies National Institute of Health research (RePORT database) Projects funded by health foundations
Not Recommended	Varies	Evidence of effectiveness is conflicting or of poor quality or both Weak theoretical foundation Balance of benefit and harm cannot be established or evidence demonstrates that harm outweighs benefits	Varies

Sources: Brownson et al (1). Republished with permission of Annual Reviews, Inc, permission conveyed through Copyright Clearance Center, Inc. Healthy people 2020. 8.

a A pre-evaluation activity that assesses whether a program or policy can be evaluated and what the barriers to evaluation might be.

## Developing and Prioritizing Program and Policy Options (Stage 5)

Planning process organizers led staff and external partners through a systematic and transparent process to prioritize the 58 rated strategies from stage 4 and to select 10 to 12 priority strategies that CDPHE would implement. The team for stage 5 was organized into 3 committees:

- Planning committee to create and facilitate the prioritization process.
- Steering committee to select criteria and use them to prioritize strategies.
- Internal executive committee to select final priorities to implement at CDPHE.

The team used common group facilitation tools to increase collaboration and transparency of information and decision-making: creating a team charter, appointing external facilitators, sharing results from each activity on a public website, voting by public roll-call during meetings, and conducting web-based surveys between meetings. The roles of the committees and their decision-making models for this stage are described in Table 1.

The planning committee, which included 2 external facilitators, planned 4 steering committee meetings at which a consensus decision-making model would be used. This committee developed 2 surveys (Table 4), shared survey results, and posted all resources and information about the process and outcomes of this stage on the CDPHE’s prevention division’s public website (http://www.coprevent.org/search/label/OIP).

**Table 4:** Tools Developed to Rank and Select Reviewed Strategies

**Tool**	**Description**
Prioritization criteria survey	This generic version of CDPHE’s tool is used to determine which criteria the steering committee will use for the prioritization process. It provides 11 frequently discussed criterion and allows for other suggestions. http://btfy.me/6mxvbt
Agency role survey	This generic version of CDPHE’s tool collects input from the steering committee on which roles would be appropriate for the agency regarding each strategy. The tool is to be completed for each strategy and may lead to the elimination of strategies that do not align well with the agency’s mission or priorities. http://btfy.me/rkh3tw
Results from criteria and agency Role survey questions	The findings from surveys administered to steering committee members soliciting their input on criteria to prioritize among the strategies and define the state health agencies role. http://btfy.me/fr6wc4
Final Prioritization Criteria Definitions	This tool describes the final 5 criteria selected by CDPHE’s obesity steering committee to prioritize among the 58 rated strategies. . http://btfy.me/wbmd3r
Resulting list of prioritized strategies	This ranked list of the 58 strategies resulted from the meetings and decisions of the steering committee and was presented to the executive committee for action and final resolution. http://btfy.me/6q9wtv
12 State obesity priorities	This tool describes the 12 selected state priorities for obesity prevention and control, which combines 19 of the original 58 strategies, and relates them to the goal or strategy in *Accelerating Progress in Obesity Prevention: Solving the Weight of the Nation* by the Institute of Medicine. http://btfy.me/thw87d

The steering committee was charged with identifying the decision criteria and using them to prioritize the 58 strategies, which the committee did by participating in 4 meetings, reviewing information from all of the prior EBPH stages (Figure), and completing the 2 surveys. The committee adopted the following decision rule: questions were to be resolved by a 60% majority vote, with 1 vote per steering committee member. At the end of each meeting, the members gave anonymous feedback and listed concerns for the planning committee to address at the next meeting.

To reduce the steering committee’s meeting time, an initial web-based survey polled members about their preferred prioritization criteria, any additional information needed, and possible CDPHE roles in each proposed strategy. At the first meeting, the steering committee discussed the survey results and voted to select these criteria:

- Likelihood of population impact (adjusted by the level of evidence).
- Capacity to implement.
- Impact on health disparities.
- Political and community support.
- Ability to measure.

A second survey was used after criteria selection to lead the steering committee through prioritization by assigning numeric, Likert-type scores to each strategy on the basis of the 5 criteria. The values assigned to each criterion were aggregated and averaged; the average scores for each criterion were then added to create a single prioritization score for each strategy. After a lengthy discussion, the steering committee voted to weight each criterion equally. However, later the members decided that “likelihood of population impact” was partially dependent on the level of evidence for each strategy. Therefore, ratings on this criterion were adjusted by using the following formula:

(Average Rating) * (Evidence Weight) = Adjusted Average Rating

A strategy with a “promising” or “emerging” evidence level was given an evidence weight of 1, a “likely effective” strategy was weighted 1.5, and a “proven” strategy was weighted 2.

The steering committee voted to submit all 58 ranked or prioritized strategies to the executive committee for selecting 10 to 12 priorities, instead of recommending only a subset of the strategies. Steering committee members also gave the executive committee a rank ordering of the strategies associated with each setting. Through the written meeting notes and feedback cards, the steering committee offered guidance on implementation for the executive committee to consider, including CDPHE’s role, coordination with external collaborators, overlap with local public health agencies’ obesity efforts, and ways to strengthen evaluation.

The executive committee, which was responsible for selecting prioritized strategies to implement, made decisions via consensus during 9 meetings: 4 that finalized the priorities and 5 that addressed communication and implementation. Executive committee members also observed the steering committee meetings to fully understand issues and decisions.

The executive committee first reviewed the 20 top-ranked strategies, which were associated with 7 of the 8 settings. They excluded media strategies because of their lower rankings and limited state role, given inadequate resources. The executive committee looked for a primary and appropriate role for the state health department and explored whether partners were better suited to implement particular strategies. Factoring in the steering committee’s written feedback, executive committee members discussed the capacity and effectiveness of the state public health department to implement a strategy. They sought staff expertise when the state role or the outcome of the strategy appeared less defined. For worksite wellness and built environment, the committee collapsed multiple strategies into 1 approach that focused on providing technical assistance to local communities and state-level partners.

The executive committee selected 12 state priorities for obesity control and prevention that encompassed 19 of the 23 top-ranked strategies. It eliminated 4 strategies that lacked a primary role for the state public health department. Before announcing the 12 priorities, the CDPHE deputy division director, who is also on the executive committee, met individually with staff members who would be most affected. She described the benefits of focusing on 12 priorities and used facts to decrease ambiguity and to manage change (9). Then the committee designated staff leads to develop implementation teams for each priority and communicated the results to all staff and stakeholders as the end of stage 5 and the start of stage 6 (action planning).

## Lessons Learned

This systematic planning process stretched over 9 months and covered 5 of the 7 stages in the evidence-based public health framework by Brownson and others (1). CDPHE is currently implementing stages 6 and 7 of the framework. The processes described in this article involved a large number of staff members in a way that allowed them to juggle daily work and the added demands of making a significant contribution to the review and prioritization process. In all, the literature review teams looked at roughly 150 articles, ranging from systematic literature reviews to peer-reviewed journal articles to the grey literature. From this, the teams distilled the research into 58 physical activity and nutrition strategies and produced a final list of 12 priority strategies that form the cornerstone of the department’s obesity prevention and control work for the next 5 years. Collectively, this work is realistic given available resources, appropriate for a state public health agency, and derived from a systematic and scientifically based process.

Feedback gathered from participants via process evaluation forms throughout these 2 stages indicated a high level of confidence in the process and the decisions. At the end of the literature review trainings and discussions, the instructors collected feedback from participants using paper and pencil surveys to assess the capacity building components and the usefulness of the tools used. Respondents indicated that they gained confidence in their ability to search for and obtain journal articles, use the CDPHE Digital Library, critique journal articles, rate the literature, and summarize the overall level of evidence using the tool Typology for Classifying Interventions and Strategies by Level of Scientific Evidence. They explained that discussing the literature with their colleagues was one of the most helpful aspects of the training. They will use the literature review process learned during the training in future planning.

A similar survey was sent to participants after the prioritization process to assess communication, the group facilitation, organization, the usefulness of the tools, and the impact of the experience. Participants’ views of the tools were mixed. Most indicated that the facilitation was skilled, and the project was well organized, though within a short time frame. Overall, participants were satisfied with the prioritization process, believed their skills improved as a result, and were confident they could apply what they learned to their work. The majority also indicated that the selected strategies are in alignment with current initiatives under way in Colorado, are appropriate for the role of CDPHE, and are aligned with the evidence.

The entire planning process and its outcome did not occur without dissension. For example, as a result of the final decisions about priorities, a few staff members had to cease work on some projects and found themselves redirected to new work. Because staff members invest deep personal meaning in their work, some experienced this change as a loss. However, the staff revealed in the survey their overall confidence in and satisfaction with the outcome.

CDPHE was fortunate to be in a pilot library program that extends access to 100 electronic journals and to databases, such as Cochrane Library’s database, and delivers electronic articles. CDPHE’s access to literature was critical to the overall success of the process. To follow this process closely, states without such access would have to find a way to access quickly such critical resources as the full scientific literature.

The end of the prioritization stage coincided with the release of the Institute of Medicine (IOM) report *Accelerating Progress in Obesity Prevention: Solving the Weight of the Nation* (10). CDPHE found significant concordance between IOM’s strategies and the 12 Colorado priorities The CDPHE process, however, emerged not from convening senior scientific experts, but from convening front-line public health workers engaged with other ongoing projects. This result suggests that this process yielded strategies and insights on a par with those of noted authorities.

The tools and approaches that CDPHE developed and used can be replicated. Other states and programs can use the same tools to implement 2 challenging stages of Brownson et al’s EBPH framework (1). By using them in stages 4 and 5, CDPHE

- Built or refreshed staff skills in reviewing the literature.
- Created a library of relevant literature that can be easily updated.
- Maintained or enhanced partnerships.
- Better aligned its priorities, state role, staffing, and day-to-day work with evidence of their effectiveness.
- Positioned itself well to motivate and prepare staff and teams to complete successfully the remaining stages of the Brownson framework and execute the work.

Colorado’s resulting portfolio of priority obesity strategies is grounded in science, built on a foundation of broad staff and stakeholder support and expertise, and therefore provides the greatest strategic opportunity for success.

## References

[1] Brownson RC , Fielding JE , Maylahn CM . Evidence-based public health: a fundamental concept for public health practice. Annu Rev Public Health 2009;30:175–201. 10.1146/annurev.publhealth.031308.100134 19296775

[2] Kohatsu ND , Robinson JG , Torner JC . Evidence-based public health: an evolving concept. Am J Prev Med 2004;27(5):417–21. 15556743 10.1016/j.amepre.2004.07.019

[3] Pawson R , Greenhalgh T , Harvey G , Walshe K . Realist review – a new method of systematic review designed for complex policy interventions. J Health Serv Res Policy 2005;10(1):21–34. 10.1258/1355819054308530 16053581

[4] Brownson RC , Baker EA , Leet TL , Gillespie KN , True WR . Evidence-based public health. Second edition. New York (NY): Oxford University Press; 2011.

[5] F as in fat: how obesity threatens America’s future 2011. Washington (DC): Trust for America’s Health; 2011. http://www.healthyamericans.org/reports/obesity2011/Obesity2011Report.pdf. Accessed April 23, 2013.

[6] Brennan L , Castro S , Brownson RC , Claus J , Orleans CT . Accelerating evidence reviews and broadening evidence standards to identify effective, promising, and emerging policy and environmental strategies for prevention of childhood obesity. Annu Rev Public Health 2011;32:199–223. 10.1146/annurev-publhealth-031210-101206 21219169

[7] Sackett DL , Straus SE , Richardson WS , Rosenberg W , Haynes RB . Evidence-based medicine: how to practice and teach EBM. Second edition. Edinburgh (GB): Churchill Livingstone; 2000.

[8] US Department of Health and Human Services. Developing healthy people 2020: Evidence-based clinical and public health: generating and applying the evidence. Washington (DC): Secretary’s Advisory Committee on National Health Promotion and Disease Prevention Objectives for 2020; 2010. http://www.healthypeople.gov/2020/about/advisory/EvidenceBasedClinicalPH2010.pdf. Accessed September 24, 2012.

[9] William Bridges. Managing transitions: making the most of change. Reading (MA): Addison-Wesley; 1991.

[10] Institute of Medicine. Accelerating progress in obesity prevention: solving the weight of the nation. Washington (DC): National Academy of Sciences; 2012. 10.3945/an.112.002733 PMC3648752 22983849

